# Ca^2+^/Calmodulin-Dependent Protein Kinase II Regulation by Inhibitor of RIPK3 Protects against Cardiac Hypertrophy

**DOI:** 10.1155/2022/7941374

**Published:** 2022-07-28

**Authors:** Jingjing Zhang, Jianan Qian, Ji Cao, Xue Wang, Wei Zhang, Xiaosong Gu

**Affiliations:** ^1^School of Medicine, Nantong University, Nantong, Jiangsu 226001, China; ^2^School of Pharmacy, Nantong University, Nantong, Jiangsu 226001, China; ^3^Jiangsu Province Key Laboratory of Neuroregeneration, Nantong University, Nantong, Jiangsu 226001, China

## Abstract

The activity of Ca^2+^/calmodulin-dependent protein kinase II *δ* (CaMKII *δ*) is central to the mechanisms of cardiovascular diseases. Receptor-interacting protein kinase 3- (RIPK3-) mediated necroptosis has been reported to contribute to cardiac dysfunction. However, the potential protective role of inhibition of RIPK3, a regulator of CaMKII, on cardiac hypertrophy remains unclear. The present study is aimed at investigating how the RIPK3 inhibitor GSK'872 regulates CaMKII activity and exploring its effect on hypertrophic cardiomyopathy (HCM). Wild-type (WT) and RIPK3 gene knockout (RIPK3^−/−^) mice were implanted subcutaneously with Alzet miniosmotic pumps (200 *μ*L) and perfused with angiotensin II (AMP-AngII) to induce cardiac hypertrophy. After WT mice were induced by AngII for 72 hours, they were injected with GSK'872 with an intraperitoneal (IP) dose of 6 mg/kg once a day for two weeks. After this, they were physiologically examined for Echocardiography, myocardial injury, CaMKII activity, necroptosis, RIPK3 expression, mixed lineage kinase domain-like protein (MLKL) phosphorylation, and mitochondrial ultrastructure. The results indicated that deletion of the RIPK3 gene or administration of GSK'872 could reduce CaMKII activity, alleviate oxidative stress, reduce necroptosis, and reverse myocardial injury and cardiac dysfunction caused by AngII-induced cardiac hypertrophy in mice. The present study demonstrated that CaMKII activation and necroptosis augment cardiac hypertrophy in a RIPK3-dependent manner, which may provide therapeutic strategies for HCM. RIPK3 inhibitor GSK'872 has a protective effect on cardiac hypertrophy and could be an efficacious targeted medicine for HCM in clinical treatment.

## 1. Introduction

Cardiac hypertrophy is a heart disease caused by the enlargement of myocardial cells in the absence of cell division. It is the heart's response to a variety of diseases including hypertension, mechanical load, myocardial infarction, arrhythmia, endocrine disorders, and mutations in the myocardial contractile protein gene [1, 2]. The hypertrophic adaptive response is the initial compensatory mechanism to the increase in cardiac output. Persistent myocardial hypertrophy can cause dilated cardiomyopathy, eventually leading to heart failure and even sudden death, with high mortality rates worldwide. The pathological hypertrophic development involves multiple intracellular signaling pathways, of note including the enhancement of those associated with calcium-regulated signaling [3, 4]. However, the exact mechanism underpinning cardiac hypertrophy remains unclear. Investigations into preventative therapeutic strategies represent urgent unmet medical need [5].

There are two predominant cell death mechanisms: apoptosis and necrosis. Apoptosis is regulated by cell signaling pathways, whilst conversely necrosis is passive and caused by environmental pressure. Necroptosis, a recently identified type of cell death, is characterized by increased cell volume, swelling of organelles, and perforation of cell membranes [6]. This is followed by cell disintegration, release of intracellular material, the triggering of innate and adaptive immune responses, and the elimination of necrotic cells by giant pinosomes. Receptor-interacting protein kinase 3 (RIPK3) is the dominant mediator of necroptosis, and RIPK3 expression enhancement has been reported to be a robust marker for necroptosis [7, 8]. RIPK3, along with receptor-interacting protein 1 (RIP1) and mixed lineage kinase domain-like protein (MLKL), is considered responsible for the initiation and execution of necroptosis. However, the role of necroptosis in cardiac hypertrophy is not well understood. Current studies suggest that RIPK3-dependent necrosis is associated with the occurrence of several cardiovascular diseases, such as myocardial ischemia-reperfusion injury, atherosclerosis, and diabetic cardiomyopathy. However, the role of RIPK3-mediated necroptosis in cardiac hypertrophy caused by pressure overload remains unclear.

CaMKII is a serine/threonine kinase that has various functions including regulation of key proteins involved in Ca^2+^handling, intercellular coupling, cell death, inflammation, and mitochondrial function. It is a pleiotropic signal that regulates Ca^2+^ circulation, contractile inflammation, gene expression, and cell survival in cardiomyocytes. CaMKII plays an important physiological role; continuous CaMKII activation is thought to accelerate heart failure and arrhythmia and is also a major factor in sudden cardiac death [9, 10]. In addition, excessive activation of CaMKII promotes cardiomyocyte activation signal transduction, oxidative stress, hyperglycemia, ischemic injury, and more. Therefore, the study of the pathophysiology of CaMKII-mediated apoptosis is an important subject in cardiovascular biology that could provide new targets for the treatment of disease. The latest study found that Ca^2+^-calmodulin-dependent protein kinase (CaMKII) is a new substrate of RIP3. RIPK3 directly phosphorylates CaMKII at amino acid T287 site (its autophosphorylation site) or indirectly through increasing ROS production. CaMKII is oxidized to activate CaMKII protein [11, 12]. In parallel, studies have observed that the activation of CaMKII can lead to the opening of mPTP and the death of cardiomyocytes, and others have demonstrated that RIPK3-mediated CaMKII activation is involved in myocardial necrosis and apoptotic cell death induced by ischemia and oxidative stress [13]. Therefore, clarifying the role and mechanism of CaMKII in pressure overload-induced cardiac hypertrophy will be of great significance for the prevention and treatment of cardiac hypertrophy.

CaMKII has four isoforms: *α*, *β*, *γ*, and *δ*, among which CaMKII *δ* is the predominant heart tissue. CaMKII *δ* mRNA precursor exons 14, 15, and 16 are regulated by various splicing factors of the SR protein family, giving rise to the generation of three CaMKII *δ* variants: CaMKII *δ*A, CaMKII *δ*B, and CaMKII *δ*C [6]. Functionally, the three CaMKII *δ* splice variants are distinct. Relevant studies have shown that the primary role of CaMKII *δ*A is in mediating myocardial excitation-contraction coupling, whilst CaMKII *δ*B is distributed primarily in the nucleus and CaMKII *δ*C is central to modification of a variety of Ca^2+^ regulatory proteins through phosphorylation. CaMKII *δ* plays an important role in the coupling of myocardial excitation and contraction. The alternative splicing of CaMKII *δ* is strictly regulated. An imbalance in the splicing of CaMKII *δ* can cause cardiomyocyte dysfunction and eventually lead to heart disease. Recent studies have reported that in the myocardial tissue of high-glucose-driven diabetic cardiomyopathy mice undergoing CaMKII *δ* alternative splicing disorder, inhibiting RIPK3 can alleviate CaMKII *δ* dysfunction in cardiomyocytes [5, 14, 15]. However, whether CaMKII *δ* alternative splicing also plays a similar role in myocardial hypertrophy is still unknown.

As an important member of the renin-angiotensin system, angiotensin II is significant in myocardial hypertrophy and remodeling. Previously, angiotensin II was reported to be predominantly involved in the regulation of blood pressure and blood volume. Angiotensin II drives the constriction of blood vessels and stimulates the secretion of aldosterone, temporarily regulating cardiovascular homeostasis [16, 17]. More recent studies have revealed that angiotensin II is a growth factor for vascular cells and cardiomyocytes; it induces tissue hypertrophy and interstitial fibrosis. Although lowering blood pressure is beneficial to myocardial hypertrophy itself, clinical trial analysis indicates that angiotensin-converting enzyme inhibitors are more effective than other antihypertensive drugs in reducing myocardial hypertrophy. This suggests that reversing myocardial hypertrophy is more strongly influenced by angiotensin II changes than the lowering blood pressure [18, 19].

Therefore, in this study, we studied the mechanism by which the RIPK3 inhibitor GSK'872 had a protective effect on myocardial hypertrophy by the regulation of CaMKII. The dysfunctional myocardium was constructed by micropump infusion of angiotensin II in RIPK3^−/−^ mice and wild-type mice. Intraperitoneal injections of RIPK3 inhibitors were given to the WT mouse and extensive examination of hypertrophic cardiac features were performed to explore the effect of RIPK3 deletion and downregulation on myocardial hypertrophy. Furthermore, the present study attempted to reveal whether silencing and downregulating of RIPK3 can regulate CaMKII *δ* alternative splicing and CaMKII activity to delay the pathogenesis of cardiac hypertrophy.

## 2. Materials and Methods

### 2.1. Animals

Male 8-week-old C57BL/6 mice (wild-type (WT)) were provided by the Experimental Animal Center of Nantong University (Nantong, China). RIPK3 knockout (RIPK3^−/−^) mice with C57BL/6 background were donated by the Institute of Molecular Medicine, Peking University (Beijing, China). The animals were kept in an SPF animal room, fed with sterilized feed and sterilized water, and the room was given a 12-hour light/dark cycle. All procedures comply with the recommendations of the “Guidelines for the Care and Use of Laboratory Animals” (approval number: NTU-20161225) issued by the National Institutes of Health and the Animal Care and Use Steering Committee of Nantong University. Animal Experimental Ethical Committee of Nantong University approved the study on the animal model (ethical clearance number: S2021224-079).

### 2.2. Establishment of Mouse Models of Cardiac Hypertrophy

After 1 wk of acclimatization, mice were anesthetized by an intraperitoneal injection of 2.5% avertin (Sigma Chemical, St. Louis, MO) at a dose of 14 *μ*L/g body wt. Alzet miniosmotic pumps (AMP, model 2002, Alza, Mountain View, CA) were then implanted subcutaneously into the flank. In the Ang II-infused group(AMP-AngII), Ang II (Sigma Chemical) was dissolved in sterile PBS containing 0.01 mol/L acetic acid and infused through the miniosmotic pumps at a rate of 2.5 *μ*g·kg^−1^·min^−1^. PBS was infused at the same rate for the control group. The infusions lasted for 2 wk. Mice were randomly divided into different groups: WT mice with PBS-infused group WT (AMP-PBS), WT mice with Ang II-infused group WT (AMP-AngII), RIPK3 KO with PBS-infused group RIPK3^−/−^ (AMP-PBS), and RIPK3 KO with Ang II-infused group RIPK3^−/−^ (AMP-AngII).

### 2.3. Administration of RIPK3 Inhibitor and Animal Treatment

RIPK3 inhibitor GSK'872 (Merck Millipore, Darmstadt, Germany) was dissolved in DMSO (<0.1%) and diluted in normal saline (NS). Wild-type (WT) and the RIPK3 gene knockout (RIPK3^−/−^) mice were implanted subcutaneously into the back with Alzet miniosmotic pumps (200 *μ*L) and perfused with angiotensin II (AMP-AngII) to induce cardiac hypertrophy. After WT mice were induced by AngII for 72 hours, they were injected intraperitoneally (IP) by GSK'872 at a dose (6 mg/kg IP) once a day for two weeks. After 2 weeks, 4 experimental groups (6 mice per group) were set up: (1) in the PBS-infused group, mice were perfused through PBS; (2) in the AngII-infused group, mice were induced by AngII diluted in PBS at a concentration (13.5 mg/ml); (3) in the AngII-infused group+NS, AngII-infused mice were injected intraperitoneally by NS; and (4) in the AngII-infused group+GSK'872 treatment, AngII-infused mice were injected by GSK'872 at a dose of 6 mg/kg IP.

### 2.4. Echocardiography

Two weeks after the micropump perfusion, mice were anesthetized with isoflurane (1-2%). After carefully clipping the hair on the left chest, cardiac geometry was measured along a parasternal long-axis section using a small animal color ultrasound system (Visual Sonic Vevo 2100, Toronto, Ontario, Canada) at a probe frequency of 30 MHz. Clear images of the left ventricular region were recorded using M-mode echocardiography. In addition, measurements of the thickness of the interventricular septum (IVS) and left ventricular posterior wall (LVPW) were recorded. Ejection fraction (EF) and left ventricular fractional shortening (FS) were then calculated as an average for 10 cardiac cycles.

### 2.5. Heart Index Determination

The blood of mice was collected by bleeding from the eyeball, and serum samples were obtained after static centrifugation. After blood collection, mice were euthanized with an overdose of isoflurane (5%). The heart was immediately excised and washed with precooled saline to remove any blood clots. All connective tissue and blood vessels attached to the heart were removed. The hearts were dried with a filter paper and then weighed with an electronic balance (HW). Left ventricular weight (LVW), including interventricular septal weight, was determined after removal of the atrium and right ventricle. Heart mass index (HMI) and left ventricular mass index (LVMI) were calculated as the ratios of HW to body weight (BW) and LVW to BW, respectively. Tibial length (TL) was measured from the edge of the tibial plateau to the medial malleolus of the right hind limb. The ratio of LVW to TL was calculated and expressed as an indicator of cardiac hypertrophy. After the measurement, the left ventricular tissue was quickly put into a -80°C low-temperature refrigerator for storage.

### 2.6. Hematoxylin-Eosin (HE) Staining

The left ventricle was fixed with 4% paraformaldehyde overnight, embedded in paraffin, and then cut into 5 *μ*m thick sections. Sections were sequentially stained with hematoxylin and eosin and dehydrated with ethanol. Finally, the sections were observed under an optical microscope and photographed.

### 2.7. Sirius Scarlet Staining

The left ventricle was fixed with 4% paraformaldehyde overnight, embedded in paraffin, and then cut into 5 *μ*m thick sections. The samples were stained with Wiegert iron hematoxylin staining solution and then stained with Sirius red staining using a standard approach involving dehydration until transparency, mounting with neutral gum, and photographing using a microscope.

### 2.8. Masson Staining

The left ventricle was fixed with 4% paraformaldehyde overnight, embedded in paraffin, and then cut into 5 *μ*m thick sections. The paraffin sections were dewaxed in water, treated with potassium dichromate chromate overnight, stained with iron hematoxylin, Ponceau acid fuchsin, phosphomolybdic acid, and aniline blue in turn, before being dehydrated and mounted for microscopic examination.

### 2.9. WGA Staining

The left ventricle was fixed with 4% paraformaldehyde overnight, embedded in paraffin, and then cut into 5 *μ*m thick sections. Paraffin sections were dewaxed in water. After antigen retrieval, a WGA working solution was added dropwise, before in incubation for 30 minutes. Samples were stained for nuclear sealing before microscopic examination.

### 2.10. TUNEL Staining

The left ventricles were fixed overnight in 4% paraformaldehyde, embedded in paraffin, and then cut into 5 *μ*m sections. After staining with TUNEL (Beyotime, Shanghai, China) at 37°C for 60 min, the sections were washed three times with PBS, before being observed and photographed using an optical microscope. Quantification was performed using ImageJ software.

### 2.11. Measurement of Superoxide Formation

Superoxide production in myocardial tissue was detected by fluorescence microscopy using the fluorescent probe dihydroethidium (DHE). Myocardial tissue sections (5 *μ*m) were prepared and subsequently incubated (30 min, 37°C) in Krebs-HEPES buffer (mM components: NaCl 99, KCl 4.7, MgSO_4_ 1.2, KH_2_PO_4_ 1.0, CaCl_2_ 1.9, NaHCO_3_ 25, glucose 11.1, NaHEPES 20; pH 7.4) and DHE (2 *μ*M) in a dark room. The slides were examined using a Nikon TE2000 inverted microscope (Nikon, Tokyo, Japan) with excitation and emission wavelengths of 480 and 610 nm, respectively.

The left ventricular tissue was homogenized with precooled PBS in the ratio of 1 : 9 and then centrifuged at 4°C and 1600 rpm for 10 min to obtain the supernatant. BCA protein concentration determination kits were used to determine the protein concentration for subsequent quantitative calculation. Malondialdehyde (MDA) levels in the myocardium were measured using the thiobarbituric acid method (Beyotime) with a lipid peroxidation and assay kit. The total antioxidant capacity (T-AOC) of the myocardium was measured by the T-AOC assay kit and plasma iron-reducing capacity method (Beyotime). The activities of total SOD, Cu-Zn/SOD, and Mn-SOD in myocardium were measured using the WST-1 (2-(4-iodophenyl)-3-(4-nitrophenyl)-5-(2,4-disulfophenyl)-)-2H-tetrazole method (Beyotime).

### 2.12. Determination of Blood Biochemical Indicators

Whole blood was collected from the orbital vein of each mouse and centrifuged at 3000 g for 20 minutes before sacrifice. Serum was stored at −80°C for other analyses. The activity of LDH and the contents of CK, IL-6, and TNF-*α* in the serum of mice were detected with corresponding commercial kits.

### 2.13. Observation of Myocardial Ultrastructure

Fresh myocardium was cut into 3 pieces of 1 mm and fixed with 4% glutaraldehyde and 1% osmic acid in the sequence. The samples were dehydrated with acetone, embedded in Epon 812, stained with toluidine blue, cut into 70 nm sections, and stained with uranyl acetate and lead citrate. The ultrastructure of myocardial tissue was examined with transmission electron microscopy (JEM-1230). In order for the mitochondrial structure and number to be assessed, visible images of 15 randomly selected regions per section at 5000x magnification were produced, and the mitochondrial volume and count were calculated.

### 2.14. Real-Time PCR

The total RNA of the myocardium was extracted from the myocardium using a Trizol separation reagent. The cDNA was then synthesized by the Prime Script™ RT Master Mix Kit (Takara, Kyoto, Japan) before quantitative real-time PCR was performed using the SYBR Green Fast qPCR mix (Takara) with the ABI StepOne PCR System (ABI, Carlsbad, CA, USA). All primers used were the following: atrial natriuretic peptide (ANP)—F 5′-GAGAAGATGCCGGTAGAAGA-3′; ANP—R 5′-AAGCACTGCCGTCTCTCAGA-3′; brain natriuretic peptide (BNP)—F 5′-CTGCTGGAGCTGATAAGAGA-3′; BNP—R 5′-TGCCCAAAGCAGCTTGAGAT-3′; CaMKII *δ*A—F 5′-CGAGAAATTTTTCAGCAGCC-3′; CaMKII *δ*A—R 5′-ACAGTAGTTTGGGGCTCCAG-3′; CaMKII *δ*B—F 5′-CGAGAAATTTTTCAGCAGCC-3′; CaMKII *δ*B—R 5′-GCTCTCAGTTGACTCCATCATC-3′; CaMKII *δ*C—F 5′-CGAGAAATTTTTCAGCAGCC-3′; CaMKII *δ*C—R 5′-CTCAGTTGACTCCTTTACCCC-3′; 18S—F 5′-AGTCCCTGCCCTTTGTACACA-3′, 18S—R 5′-CGATCCGAGGGCCTCACTA-3′.

We normalized the experimental period threshold to 18S, a housekeeping gene, and calculated relative mRNA expression relative to control samples.

### 2.15. Western Blotting

Protein samples isolated from cardiac tissue were separated by SDS-PAGE and transferred onto PVDF membranes (Millipore, Billerica, MA, USA). After blocking with TBST buffer (Tris–HCl 10 mmol·L-1, NaCl 120 mmol·L-1, in 5% *v*/*v* skimmed milk, Tween-20 0.1%; pH 7.4) for 2 hours at room temperature, the membranes were incubated with the appropriate amount of primary antibody at 4°C overnight. The main primary antibodies are as follows: anti-ox-CaMKII (1 : 1000) (Millipore, Kenilworth, NJ, USA); anti-CaMKII (1 : 1000) (Abcam, Cambridge, UK); anti-caspase3, anti-cleaved caspase 3, anti-MLKL, anti-p-MLKL, and anti-RIPK1 (1 : 1000, Cell Signaling Technology, Danvers, MA, USA); anti-p-CaMKII (1 : 1000, Thermo Fisher Scientific, Rockford, IL, USA); and anti-GAPDH (1 : 5000, Sigma-Aldrich, St. Louis, MO, USA). Blots were then incubated with horseradish peroxidase- (HRP-) conjugated secondary antibody for 1.5 hours at room temperature. Protein bands were visualized by enhanced chemiluminescence (ECL) (Thermo Fisher Scientific, Rockford, IL, USA). GAPDH or *β*-tubulin was used as the loading control.

### 2.16. Statistical Analysis

All data are presented as the standard error of the mean. GraphPad Prism software was used to process the experimental data. Statistical analysis was performed by the unpaired Student's *t*-test on comparisons between two groups and by the one-way ANOVA test followed by the Student-Newman-Keuls (SNK) test on comparisons among multiple groups. *P* < 0.05 was considered statistically significant.

## 3. Results

### 3.1. Angiotensin (AngII) Induces Cardiac Hypertrophy in Mice, Leading to Cardiac Dysfunction

**Figure 1:**
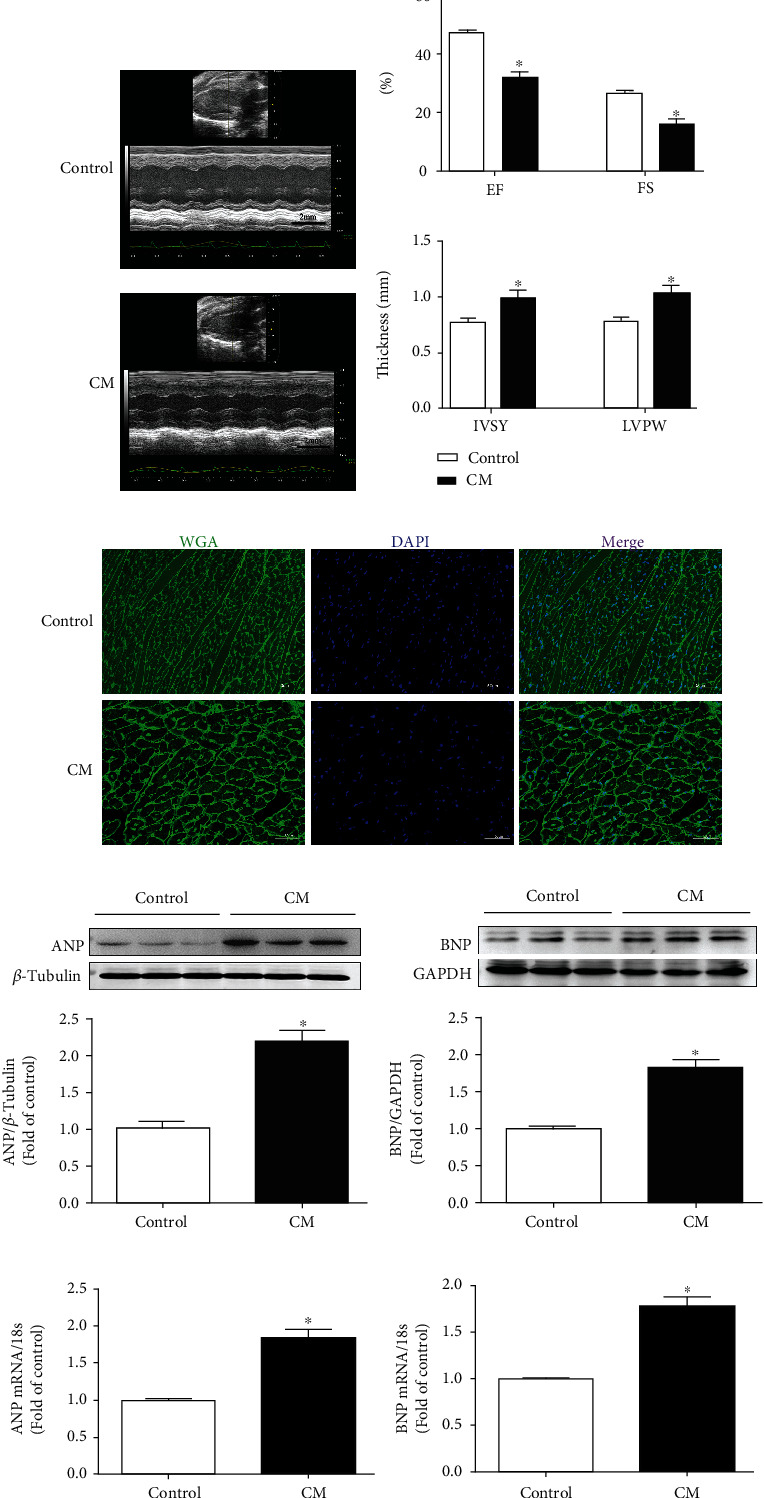
Two weeks after the subcutaneous implantation of micropump-AngII (AMP-AngII) in wild-type (WT) mice, cardiac function was assessed by echocardiography, ejection fraction (EF), short-axis shortening rate (FS), interventricular septum thickness (IVS), and left ventricular posterior wall thickness (LVPW) were calculated. Bar = 2 mm. (a, b) Cardiomyocyte hypertrophy was observed by WGA staining. Bar = 50 *μ*m. (c) Myocardial ANP and BNP protein expression was quantified by western blot. (d, e) Real-time quantitative PCR was used to detect the expression of ANP mRNA and BNP mRNA of cardiac hypertrophy genes (f, g), compared with the control (AMP-PBS) group. ^∗^*P* < 0.05; the difference was significant; *n* = 6.

Echocardiography of wild-type mice revealed reduced systolic and diastolic functions in those with angiotensin- (AngII-) induced myocardial hypertrophy; as such, associated cardiac ejection fraction (EF) and short-axis shortening (FS) were significantly decreased (*P* < 0.05) compared to mice in the control group (Figures 1(a) and 1(b)). WGA staining revealed that the cross-sectional area of myocardial cells, interventricular septum thickness (IVS), and left ventricular posterior wall thickness (LVPW) were significantly increased (*P* < 0.05) (Figures 1(b) and 1(c)). Furthermore, the expression of ANP and BNP hypertrophy genes ANP mRNA and BNP mRNA in wild-type mice were significantly increased (*P* < 0.05) (Figures 1(d)–1(g)). Overall, this indicated a myocardial hypertrophy model was successfully established in the mice in this study.

### 3.2. Overexpression of RIPK3 in Mice with Cardiac Hypertrophy Mediates CaMKII *δ* Alternative Splicing Disorder, Leading to Cardiomyocyte Necroptosis

**Figure 2:**
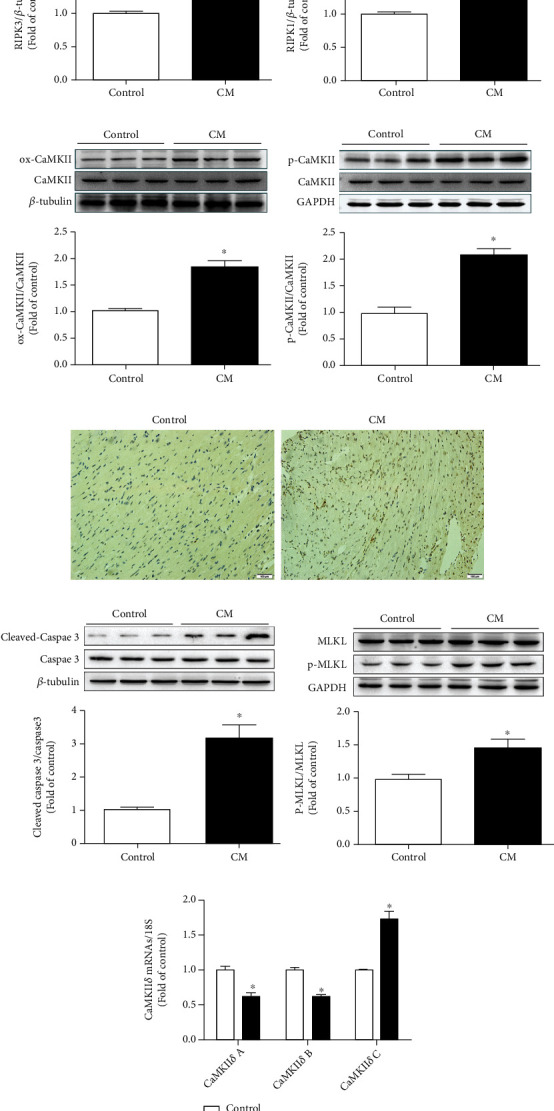
Two weeks after the subcutaneous implantation of minipump-AngII (AMP-AngII) in wild-type (WT) mice, myocardial tissue in RIPK3, RIPK1, ox-CaMKII, p-CaMKII, MLKL, P-MLKL, cleaved-caspase3, and caspase3 protein expression were quantified by western blot (a–d, f, g). Apoptosis of myocardial tissue was observed by TUNEL staining. Bar = 100 *μ*m (e). The mRNA expression of myocardial CaMKII *δ*A, CaMKII *δ*B, and CaMKII *δ*C was detected by real-time quantitative PCR (h), compared with the control (AMP-PBS) group. ^∗^*P* < 0.05; the difference was significant; *n* = 6.

Our study verified that levels of RIPK3 and RIP1 were significantly increased in the hearts of angiotensin- (AngII-) induced cardiac hypertrophy in wild-type mice (*P* < 0.05), as shown in Figures 2(a)and 2(b)). In these mice, CaMKII *δ* oxidation and phosphorylation were high, and the expressions of ox-CaMKII and p-CaMKII were significantly increased (*P* < 0.05) (Figures 2(c) and 2(d)). Disorder of CaMKII *δ* alternative splicing promotes cardiomyocyte dysfunction and ultimately heart disease [11, 13]. Since no specific antibodies for CaMKII *δ* variants were available, CaMKII *δ*A, CaMKII *δ*B, and CaMKII *δ*C mRNA expression levels were measured by quantitative real-time PCR. There was a significant decrease in CaMKII *δ*A and CaMKII *δ*B (*P* < 0.05) expression, whilst CaMKII *δ*C expression was upregulated in cardiac hypertrophy mice (*P* < 0.05) indicating disorder of CaMKII *δ* alternative splicing in mice with CM (Figure 2(h)). Since apoptosis is a critical manifestation of necroptosis, TUNEL staining showed that myocardial cell necrosis in mice was significantly increased, as were the expression levels of MLKL, P-MLKL, cleaved-caspase3, and caspase3 (*P* < 0.05) (Figures 2(e) and 2(g)).

### 3.3. RIPK3 Deficiency Alleviates Cardiac Dysfunction and Attenuates Myocardial Injury in Cardiac Hypertrophy Mice

**Figure 3:**
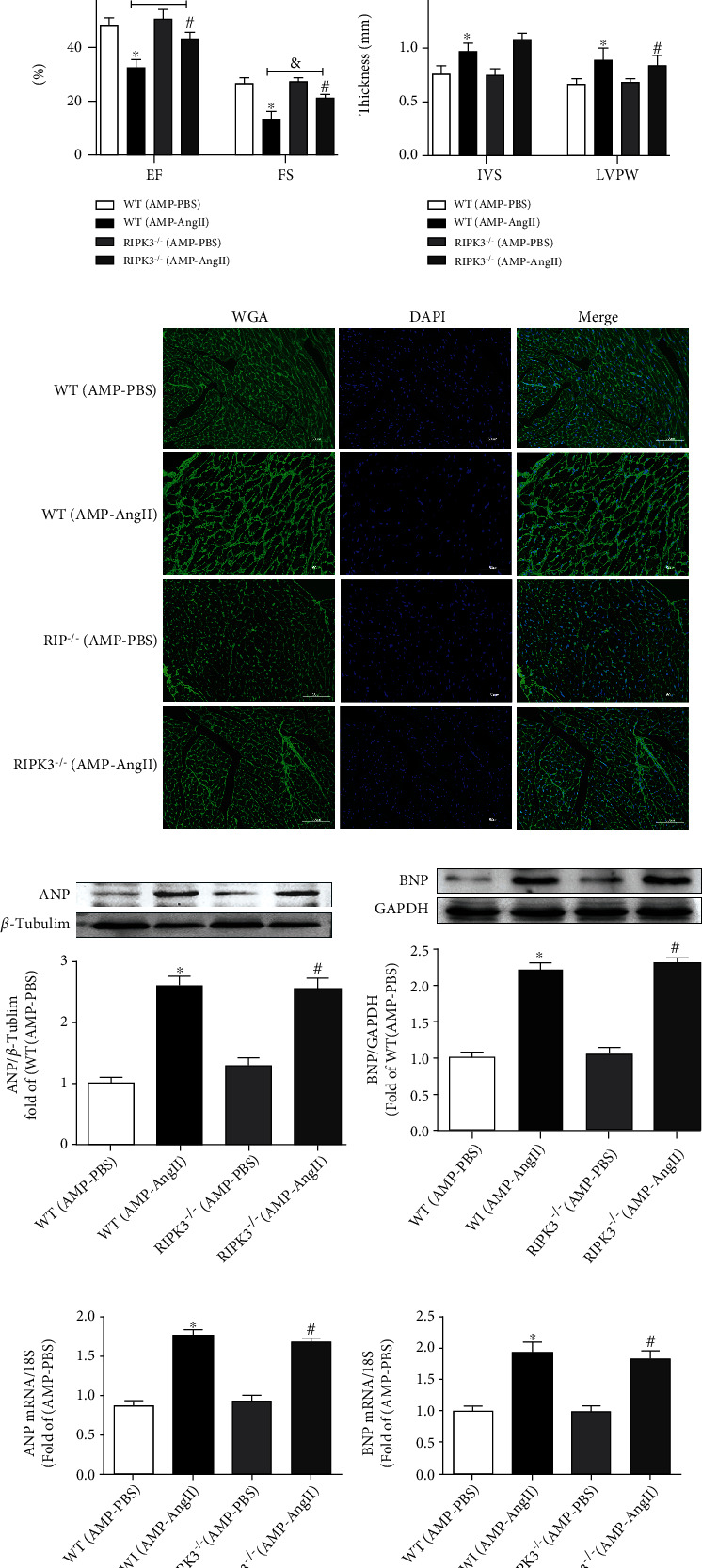
RIPK3 knockout mice (RIPK3^−/−^) and wild-type (WT) mice were subcutaneously implanted with micropump-AngII (AMP-AngII) two weeks after surgery; cardiac function was assessed by echocardiography. Bar = 2 mm (a); ejection fraction (EF), short-axis shortening rate (FS), interventricular septum thickness (IVS), and left ventricular posterior wall thickness (LVPW) (b–c) were calculated. Cardiomyocyte hypertrophy was observed by WGA staining. Bar = 50 *μ*m (d). Myocardial ANP and BNP protein expression was quantified by western blot (e, f); real-time quantitative PCR was used to detect the expression of ANP mRNA and BNP mRNA of cardiac hypertrophy genes (g, h); the WT (AMP-AngII) group compared with the WT (AMP-PBS) group, ^∗^*P* < 0.05; the RIPK3^−/−^(AMP-AngII) group compared with the RIPK3^−/−^(AMP-PBS) group, ^#^*P* < 0.05; compared with the WT (AMP-AngII) group, ^&^*P* < 0.05; the difference was significant; *n* = 6.

To further investigate the contribution of RIPK3 to the development of DCM, RIPK3-KO (RIPK3^−/−^) mice were investigated. Echocardiography was performed for RIPK3-KO (RIPK3^−/−^) mice with myocardial hypertrophy, and it was observed that the cardiac systolic and diastolic functions were significantly improved when compared to the WT (AMP-AngII) group; cardiac ejection fraction (EF) and short-axis shortening rate (FS) were significantly increased (*P* < 0.05) (Figures 3(a) and 3(b)). WGA staining was used to examine the cross-sectional area of mouse cardiomyocytes. Compared with the WT (AMP-AngII) group, the thickness of the interventricular septum (IVS) and that of the left ventricular posterior wall (LVPW) were not significantly altered (*P* > 0.05) (Figures 3(b) and 3(c)). Additionally, myocardial ANP, BNP hypertrophy gene ANP mRNA, and BNP mRNA expression in RIPK3^−/−^ mice were significantly increased when compared with the WT (AMP-PBS) group (*P* < 0.05) but not when compared with the WT (AMP-AngII) group. Sexual differences (*P* > 0.05) are shown in Figures 3(d)–3(g).

### 3.4. RIPK3 Deficiency in Myocardial Hypertrophic Mice Regulates CaMKII *δ* Splicing Disorder and Inhibits Myocardial Necroptosis

**Figure 4:**
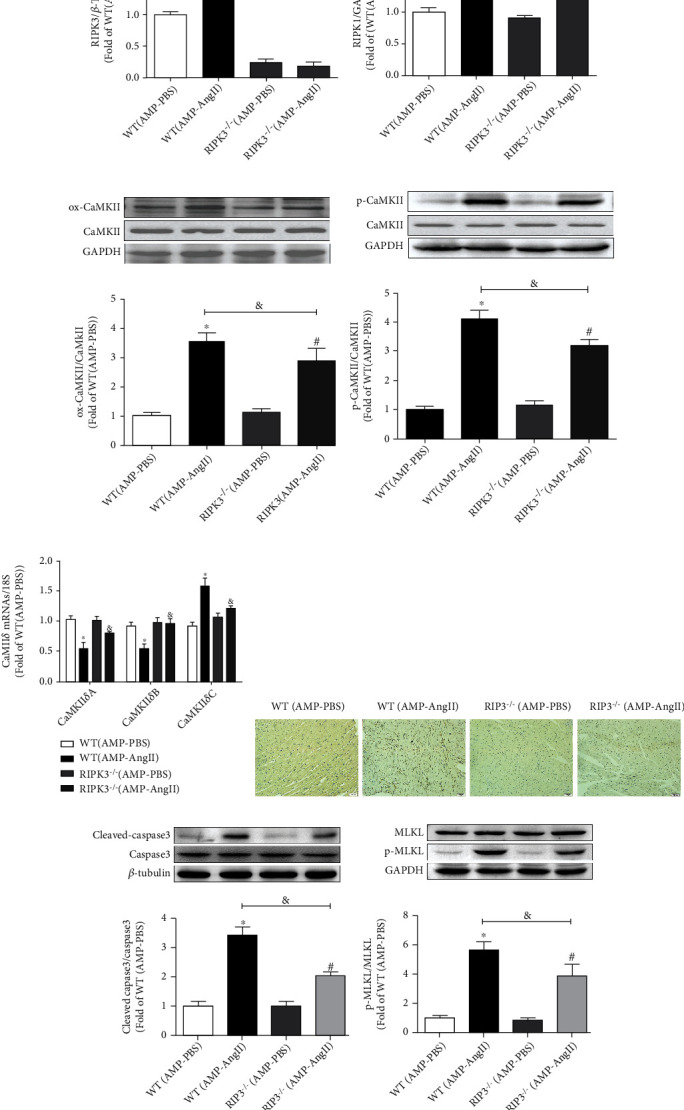
RIPK3 knockout mice (RIPK3^−/−^) wild-type (WT) were mice subcutaneously implanted with micropump-AngII (AMP-AngII) two weeks after surgery, myocardial tissue in RIPK3, RIPK1, ox-CaMKII, p-CaMKII, MLKL, P-MLKL, cleaved-caspase3, and caspase3 protein expression were quantified by western blot (a–d, g, h). Apoptosis of myocardial tissue was observed by TUNEL staining. Bar = 100 *μ*m (f). The mRNA expressions of cardiac CaMKII *δ*A, CaMKII *δ*B, and CaMKII *δ*C were detected by real-time quantitative PCR (e). The WT (AMP-AngII) group compared with the WT (AMP-PBS) group, ^∗^*P* < 0.05; the RIPK3^−/−^(AMP-AngII) group compared with the RIPK3^−/−^(AMP-PBS) group, ^#^*P* < 0.05; compared with the WT (AMP-AngII) group, ^&^*P* < 0.05; the difference was significant; *n* = 6.

In AngII-induced cardiac hypertrophy RIPK3^−/−^ mice, there was no significant expression of RIPK3 protein (*P* > 0.05) when compared with the WT (AMP-AngII) group. In addition to RIPK3, studies have reported that RIPK1 is involved in the necrosis process. RIPK1 levels were also significantly decreased (*P* < 0.05) in RIPK3^−/−^ mice, as shown in Figures 4(a) and 4(b). Further, CaMKII *δ* oxidation and phosphorylation levels were decreased in RIPK3^−/−^ mice compared with the WT (AMP-AngII) group, and the expression levels of ox-CaMKII and p-CaMKII were significantly reduced (*P* < 0.05) (Figures 4(c)and 4(d)). However, the expression of CaMKII *δ* splice variants CaMKII *δ*A, CaMKII *δ*B, and CaMKII *δ*C was significantly increased (*P* < 0.05) in RIPK3^−/−^ mice compared to WT, as shown in Figure 4(h). TUNEL staining revealed that myocardial cell necrosis in RIPK3^−/−^ mice was significantly reduced, and the expression levels of MLKL, P-MLKL, cleaved-caspase3, and caspase3 were all significantly reduced compared with the WT (AMP-AngII) group (*P* < 0.05), as shown in Figures 4(e)–4(g)).

### 3.5. RIPK3 Deficiency in Mice with Myocardial Hypertrophy Inhibits Myocardial Fibrosis and Inflammation

**Figure 5:**
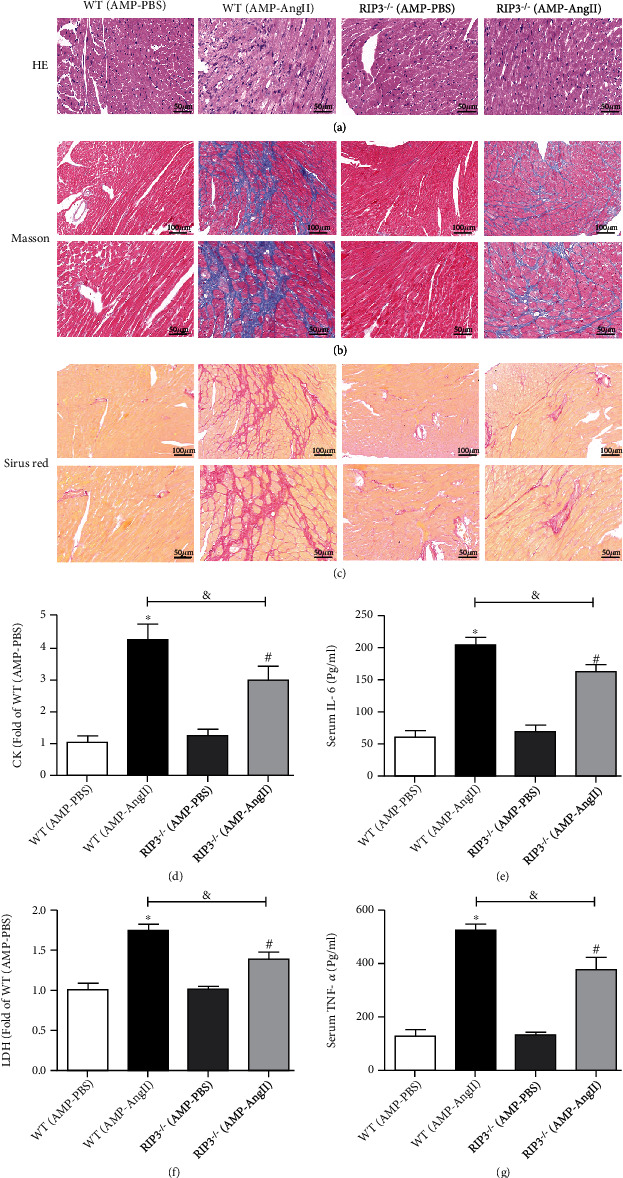
RIPK3 knockout mice (RIPK3^−/−^) and wild-type (WT) mice were subcutaneously implanted with micropump-AngII (AMP-AngII) two weeks after surgery, the mouse hearts were taken for histopathological examination, and HE were performed, respectively. Staining: bar = 50 *μ*m (a), Masson staining: bar = 100 *μ*m [upper] and bar = 50 *μ*m [lower] (b), and Sirius Red staining: bar = 100 *μ*m [upper] and bar = 50 *μ*m [lower] (c) were used to observe the distortion and disorder of myocardial tissue cells and to improve the degree of myocardial injuries such as collagen deposition and myocardial fibrosis. Serum of mice was taken to measure lactate dehydrogenase (LDH) activity, creatine kinase (CK) activity, inflammatory factor interleukin-6 (IL-6), and tumor necrosis factor (TNF-*α*) levels (d–g). The WT (AMP-AngII) group compared with the WT (AMP-PBS) group, ^∗^*P* < 0.05; the RIPK3^−/−^(AMP-AngII) group compared with the RIPK3^−/−^(AMP-PBS) group, ^#^*P* < 0.05, compared with the WT (AMP-AngII) group, ^&^*P* < 0.05; the difference was significant; *n* = 6.

During HE staining, it was observed that RIPK3^−/−^ alleviated the distortion of myocardial cells in myocardial hypertrophy mice, as shown in Figure 5(a). Masson staining and Sirius Red staining showed that the size of myocardial cells of RIPK3^−/−^ mice was significantly reduced, improving collagen deposition and myocardial fiber structure (Figures 5(b)an 5(c)). The levels of serum CK, LDH, IL-6, and TNF-*α* were significantly lower in RIPK3^−/−^ mice than in those in the WT (AMP-AngII) group (*P* < 0.05), as shown in Figures 4(d)–4(g).

### 3.6. RIPK3 Deficiency in Mice with Myocardial Hypertrophy Improves Oxidative Stress and Mitochondrial Ultrastructure in Cardiomyocytes

**Figure 6:**
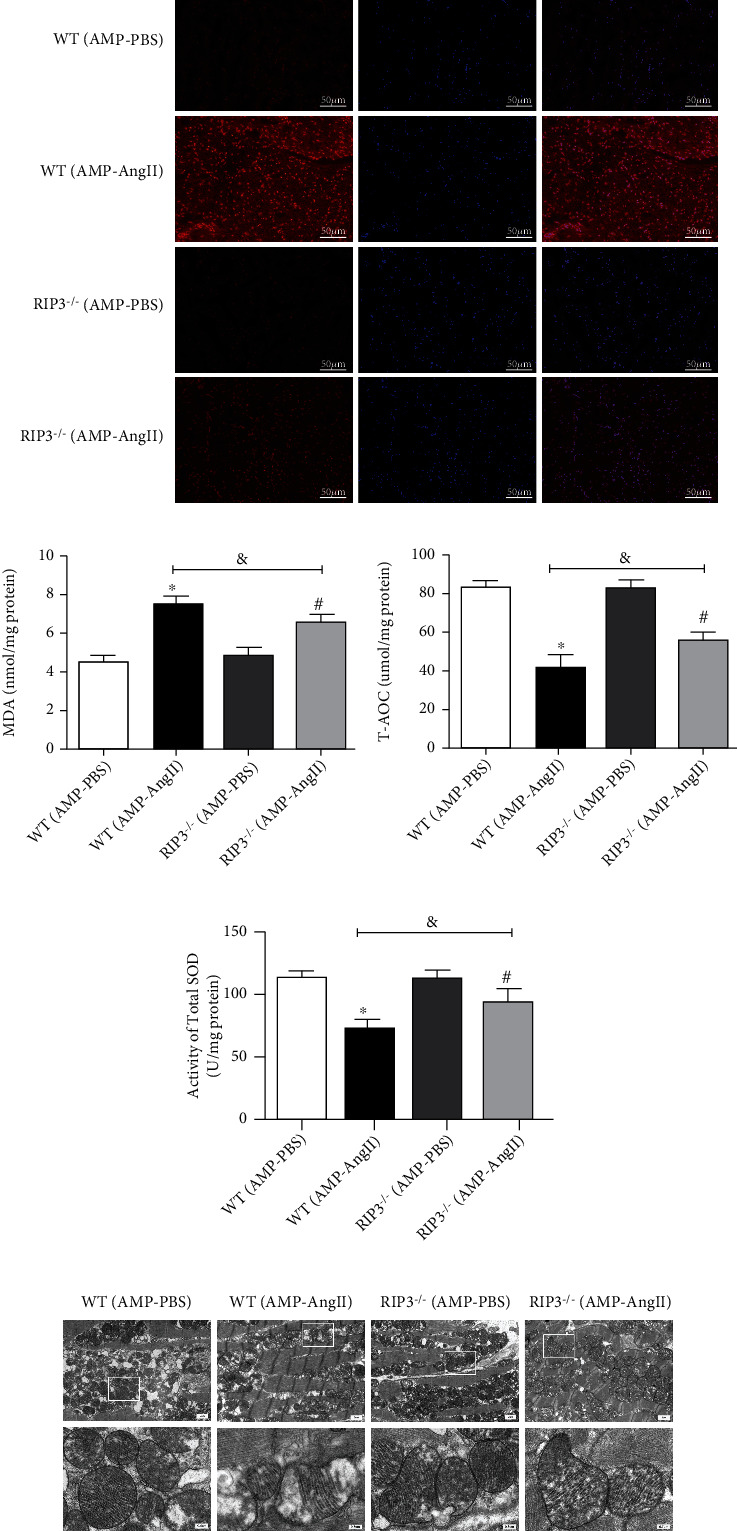
RIPK3 gene knockout mice (RIPK3^−/−^) wild-type (WT) mice were subcutaneously implanted with micropump-AngII (AMP-AngII) two weeks after surgery, the mouse hearts were taken for histopathological examination, and DHE was performed, respectively. Staining: bar = 50 *μ*m (a); the transmission electron microscope was used to observe the ultrastructure of left ventricular mitochondria; bar = 2 *μ*m [upper] and bar = 0.5 *μ*m [lower] (e); mouse serum was taken to measure superoxide dismutase (SOD) activity, malondialdehyde (MDA), and total antioxidant capacity (T-AOC) levels (b–d); the WT (AMP-AngII) group compared with the WT (AMP-PBS) group, ^∗^*P* < 0.05; the RIPK3^−/−^(AMP-AngII) group compared with the RIPK3^−/−^(AMP-PBS) group, ^#^*P* < 0.05; compared with the WT (AMP-AngII) group, ^&^*P* < 0.05; the difference was significant; *n* = 6.

DHE staining revealed an increase in red fluorescence intensity in myocardial tissue of WT mice compared to in RIPK3^−/−^ mice. Furthermore, the levels of ROS in RIPK3^−/−^ mice were reduced, whilst oxidative stress was increased, as observed by several measurements as follows: the ability of myocardial tissue to scavenge oxygen free radicals was enhanced, as shown in Figure 6(a); the serum superoxide dismutase (SOD) activity, malondialdehyde (MDA) and total antioxidant capacity (T-AOC) levels were significantly higher than in the control group (*P* < 0.05), as shown in Figures 6(b)–6(d). Finally, the ultrastructure of mitochondria in the left ventricle of myocardial hypertrophy mice was observed by transmission electron microscopy, and mitochondrial irregularity, swelling, and cristae rupture were all ameliorated in RIPK3^−/−^ mice compared to myocardial hypertrophy WT mice (Figure 6(e)).

### 3.7. RIPK3 Inhibitor GSK'872 Reverses Cardiac Dysfunction and Inhibits Myocardial Injury in Hypertrophic Mice

**Figure 7:**
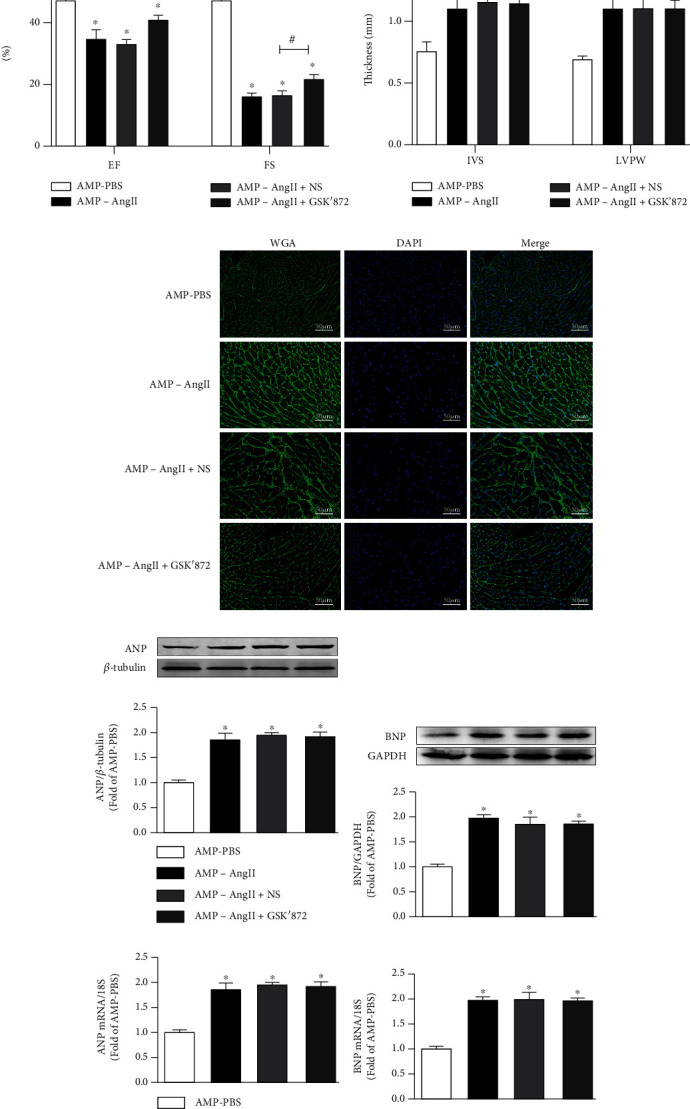
Wild-type (WT) mice were subcutaneously implanted with micropump-AngII (AMP-AngII) 72 hours after surgery and intraperitoneally injected with RIPK3 inhibitor GSK'87. Two weeks later, cardiac function was assessed by echocardiography; bar = 2 mm (a); ejection fraction (EF), short-axis shortening rate (FS), interventricular septum thickness (IVS), and left ventricular posterior wall thickness (LVPW) (b, c); cardiomyocyte hypertrophy was observed by WGA staining; bar = 50 *μ*m (d); myocardial ANP and BNP protein expression was quantified by western blot (e, f); real-time quantitative PCR was used to detect the expression of ANP mRNA and BNP mRNA of cardiac hypertrophy genes (g, h); the AMP-AngII group compared with the AMP-PBS group, ^∗^*P* < 0.05; the AMP-AngII+GSK'872 group compared with the AMP-AngII+NS group, ^#^*P* < 0.05; compared with the AMP-AngII group, ^&^*P* < 0.05; the difference was significant; *n* = 6.

The echocardiogram of mice in Figure 6(a) illuminates that the contraction, relaxation, and overall cardiac function of mice with AngII-induced myocardial hypertrophy were reduced. Compared with those in the WT (AMP-PBS) group, the cardiac EF and FS of the AngII-induced myocardial hypertrophy mice in each group were significantly decreased (*P* < 0.05), as shown in Figure 7(b). By WGA staining, it was observed that in the cross-sectional area of mouse cardiomyocytes the IVS and LVPW of each myocardial hypertrophy mouse group were significantly increased (*P* < 0.05), compared with WT (AMP-AngII)+NS group given RIPK3 inhibitor GSK'872 (Figures 7(c) and 7(d)). Further, EF and FS were significantly increased (*P* < 0.05), as shown in Figure 6(b). The levels of expression of myocardial ANP and BNP hypertrophy genes (ANP mRNA and BNP mRNA) of mice in the group were significantly increased (*P* < 0.05), as shown in Figures 7(e)–7(h).

### 3.8. RIPK3 Inhibitor GSK'872 Corrects CaMKII *δ* Alternative Splicing Disorder and Inhibits Cardiomyocyte Necroptosis in Cardiac Hypertrophic Mice

**Figure 8:**
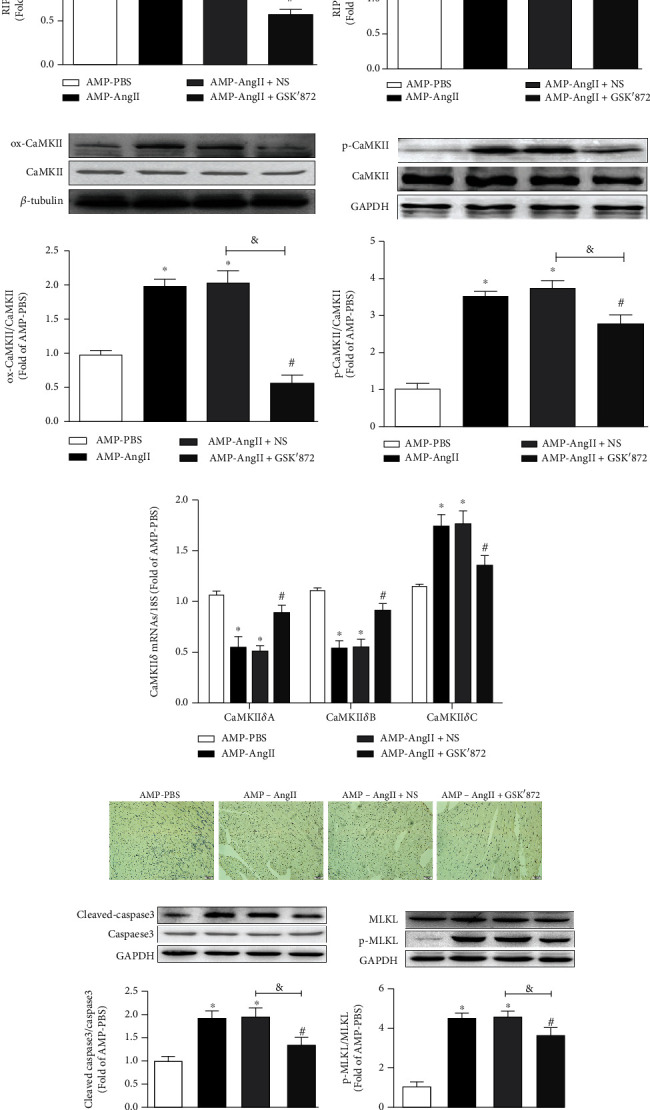
Wild-type (WT) mice were subcutaneously implanted with micropump-AngII (AMP-AngII) 72 hours after surgery and intraperitoneally injected with RIPK3 inhibitor GSK'872. Two weeks later, myocardial tissue in RIPK3, RIPK1, ox-CaMKII, p-CaMKII, MLKL, P-MLKL, cleaved-caspase3, and caspase3 protein expression were quantified by western blot (a–d, g, h); Apoptosis of myocardial tissue was observed by TUNEL staining. Bar = 100 *μ*m (f). The mRNA expressions of cardiac CaMKII *δ*A, CaMKII *δ*B, and CaMKII *δ*C were detected by real-time quantitative PCR (e). the AMP-AngII group compared with the AMP-PBS group, ^∗^*P* < 0.05; the AMP-AngII+GSK'872 group compared with the AMP-AngII+NS group, ^#^*P* < 0.05; compared with the AMP-AngII group, ^&^*P* < 0.05; the difference was significant; *n* = 6.

The expression levels of RIPK3, RIPK1, ox-CaMKII, p-CaMKII, MLKL, P-MLKL, cleaved-caspase3, and caspase3 protein in AngII-induced cardiac hypertrophy WT mice were significantly increased (*P* < 0.05) in WT (AMP-AngII) compared with the NS group. Meanwhile, the protein expression levels of RIPK3, RIPK1, ox-CaMKII, p-CaMKII, MLKL, P-MLKL, cleaved-caspase3, and caspase3 were all decreased significantly (*P* < 0.05) after administration of RIPK3 inhibitor (GSK'872) (*P* < 0.05), as shown in Figures 8(a)–8(g). Furthermore, AngII-induced cardiac hypertrophy in WT mice was associated with changes in the levels of expression of CaMKII *δ* alternative splicing variants, with CaMKII *δ*A and CaMKII *δ*B expression significantly decreasing (*P* < 0.05), whilst CaMKII *δ*C expression was significantly increased (*P* < 0.05). However, when the (AMP-AngII)+NS group was compared to those given the RIPK3 inhibitor GSK'872, the expressions of CaMKII *δ*A and CaMKII *δ*B were significantly increased (*P* < 0.05), and the expression of CaMKII *δ*C was significantly decreased (*P* < 0.05), as shown in Figure 8(h).

### 3.9. RIPK3 Inhibitor GSK'872 Attenuates Myocardial Fibrosis and Inflammation in Hypertrophic Mice

**Figure 9:**
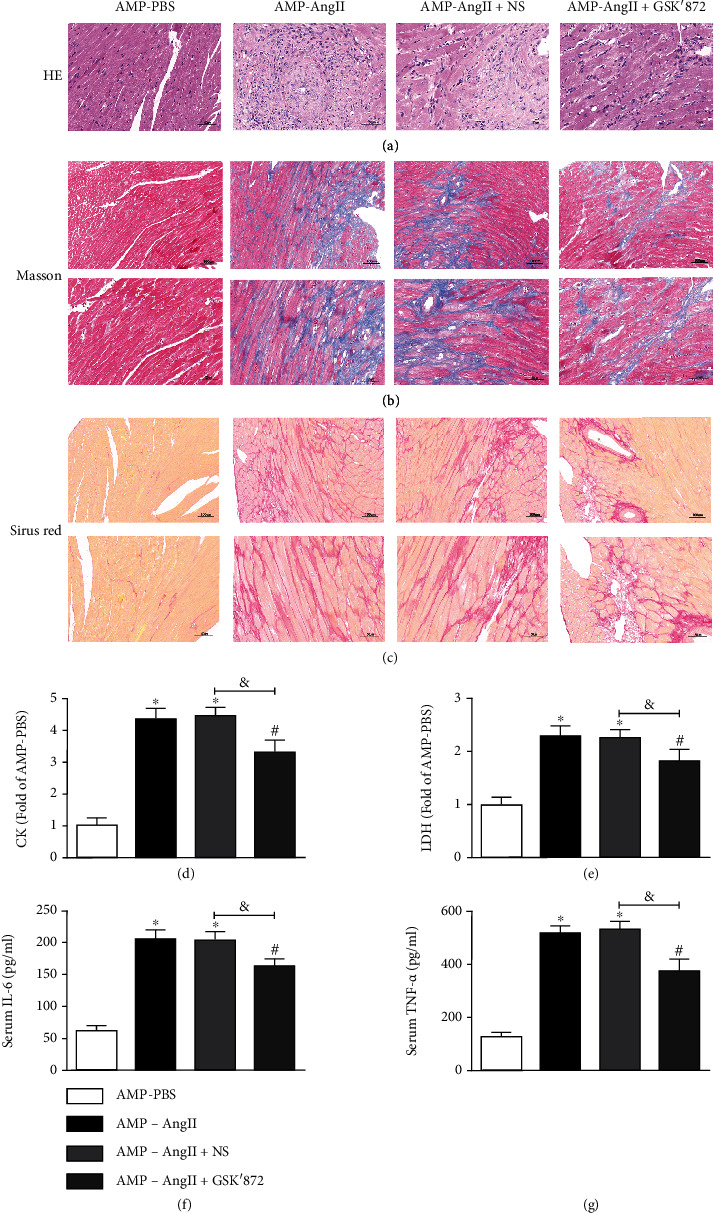
Wild-type (WT) mice were subcutaneously implanted with micropump-AngII (AMP-AngII) 72 hours after the operation, and the RIPK3 inhibitor GSK'872 was intraperitoneally injected. Two weeks later, the mouse hearts were taken for histopathological examination. HE staining: bar = 50 *μ*m, Masson staining: bar = 100 *μ*m [upper] and bar = 50 *μ*m [lower], and Sirius Red staining: bar = 100 *μ*m [upper] and bar = 50 *μ*m [lower]. (a–c) were used to observe the distortion and disorder of myocardial tissue cells and to improve the degree of myocardial damage such as collagen deposition and myocardial fibrosis; serum CK activity, LDH activity, IL-6, and TNF-*α* levels. (D-G), AMP-AngII group compared with AMP-PBS group, ∗P < 0.05; AMP-AngII + GSK'872 group compared with AMP-AngII +NS group, #P < 0.05, compared with AMP-AngII group, &P < 0.05, the difference was significant, n = 6.

HE staining revealed that the RIPK3 inhibitor GSK'872 alleviated myocardial cell distortion and disorder in myocardial hypertrophy mice (Figure 9(a)). AngII-induced myocardial hypertrophy in WT mice with increased serum CK, LDH, IL-6, and TNF-*α* levels compared with the control group. Compared with the (AMP-AngII)+NS group, the levels of CK, LDH, IL-6, and TNF-*α* were however significantly decreased when compared to those mice given the RIPK3 inhibitor GSK'872 (*P* < 0.05) (Figures 9(d)–9(g)). Masson staining and Sirius Red staining showed that the myocardium of mice treated with GSK'872 was significantly reduced to improve collagen deposition and alleviate myocardial fibrosis, as shown in Figures 9(b) and 9(c).

### 3.10. RIPK3 Inhibitor GSK'872 Improves Oxidative Stress and Myocardial Mitochondrial Ultrastructure in Mice with Cardiac Hypertrophy

**Figure 10:**
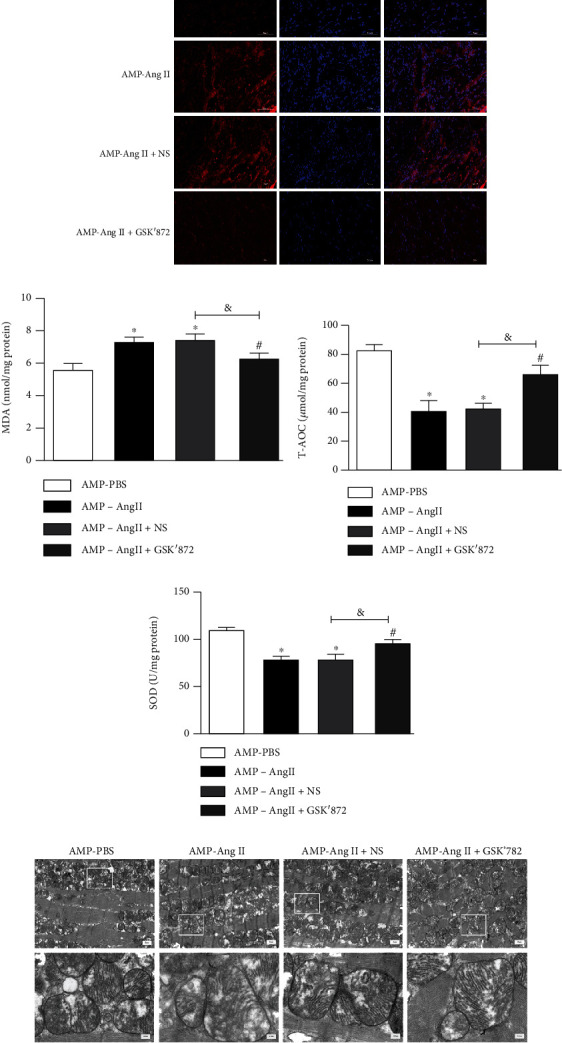
Wild-type (WT) mice were subcutaneously implanted with micropump-AngII (AMP-AngII) 72 hours after the operation and intraperitoneally injected with the RIPK3 inhibitor GSK'872. Two weeks later, the mouse hearts were taken for histopathological examination, and DHE was performed, respectively. Staining: bar = 50 *μ*m (a); the transmission electron microscope was used to observe the ultrastructure of left ventricular mitochondria; bar = 2 *μ*m [upper] and bar = 0.5 *μ*m [lower] (e); mouse serum was taken to measure SOD activity (b); MDA and T-AOC levels (b–d); the AMP-AngII group compared with the AMP-PBS group, ^∗^*P* < 0.05; the AMP-AngII+GSK'872 group compared with the AMP-AngII+NS group, ^#^*P* < 0.05; compared with the AMP-AngII group, ^&^*P* < 0.05; the difference was significant; *n* = 6.

Using DHE staining, it was observed that red fluorescence staining intensity in myocardial tissue of RIPK3 inhibitor (GSK'872)-treated mice was attenuated compared with AngII-induced myocardial hypertrophic WT mice. Further, mice treated with the RIPK3 inhibitor (GSK'872) also had lower levels of red staining intensity than in WT mice. After GSK'872 treatment, the level of ROS in mice decreased, the level of oxidative stress in mice increased, and the ability of myocardial tissue to scavenge oxygen free radicals was enhanced, as shown in Figure 10(a): AngII-induced myocardial hypertrophy WT mice had significantly higher serum MDA levels (*P* < 0.05), whilst SOD activity and T-AOC level were significantly decreased (*P* < 0.05); whilst when compared with the (AMP-AngII)+NS group, the serum MDA level was significantly decreased (*P* < 0.05) when the RIPK3 inhibitor (GSK'872) was administered and the T-AOC level was significantly increased (*P* < 0.05), as shown in Figures 10(b)–10(d)). Finally, transmission electron microscopy was used to observe the ultrastructure of left ventricular mitochondria in mice with AngII-induced myocardial hypertrophy: mitochondrial irregularity, swelling, and cristae fragmentation were all observed. However, when RIPK3 inhibitor GSK'872 was administered, the abnormal mitochondrial ultrastructure of hypertrophic cardiomyocytes in mice was significantly improved, as shown in Figure 10(e).

## 4. Discussion

Myocardial hypertrophy can include both physiological hypertrophy and pathological hypertrophy. Persistent pathological myocardial hypertrophy can lead to congestive heart failure, arrhythmia, and sudden death [20, 21]. Myocardial hypertrophy is often accompanied by cardiac dysfunction and myocardial damage. In this study, a mouse model of myocardial hypertrophy was successfully constructed by implanting angiotensin II (AMP-AngII) using a subcutaneous micropump. We identified reduced EF and FS values in the hearts of mice with myocardial hypertrophy, associated with hyperactivity in serum LDH and CK and increased levels of deposition of myocardial collagen. Furthermore, levels of MDA, T-AOC, and T-SOD in myocardial tissue were raised, and the levels of serum IL-6 and TNF-*α* were elevated. Mitochondrial dysfunction was also evident. Our findings were consistent with the pathophysiological characteristics of myocardial hypertrophy, such as myocardial fibrosis, with clear evidence of oxidative stress damage, inflammatory response, and cellular dysfunction [22, 23]. As a recently discovered mechanism of cell death, many studies have reported that necroptosis exerts a significant role in the occurrence and development of a variety of cardiovascular diseases [24, 25]. Our experiments revealed that myocardial cell necroptosis was triggered during myocardial hypertrophy in mice. Given that RIPK3 is a key signaling molecule in necroptosis-related pathways, the present study compared the differences in cardiac hypertrophy presentation between WT mice and RIPK3^−/−^ mice and mice treated with the RIPK3 inhibitor GSK'872. In doing so, we attempted to demonstrate consistency between the effects of knockout of the RIPK3 gene and administration of an RIPK3 inhibitor and reveal the efficacy of RIPK3 inhibitors in reducing myocardial damage, improving cardiac function in cardiac hypertrophy.

RIPK3 is the convergence point of a variety of signaling pathways, including those of necrosis, inflammation, and oxidative stress [26]. RIPK3 acts via interaction of its homologous RIPK1 with the RIP homotopy interaction motif (RHIM) domain of RIPK3 to promote the formation of necrosomes [27]. The formation of necrotic bodies results in the activation (phosphorylation) of MLKL which is subsequently translocated to the plasma membrane. Once localized to the plasma membrane, MLKL forms permeable pores, leading to disruption of the integrity of the plasma membrane and, subsequently, to necroptosis [28, 29]. Furthermore, tumor necrosis factor-*α* (TNF-*α*) has been reported to play a key role in the initiation of necrosis and the induction of stimulated necrosis. TNF-*α* can activate cell suicide mechanisms, such as apoptosis and necrosis, and trigger the accumulation and phosphorylation of RIPK1 and RIPK3, resulting in the phosphorylation of MLKL, the formation of necrotic bodies, and necrosis [30]. Moreover, recent studies have identified that the elevated levels of necroptosis is accompanied by an increase in pro-inflammatory cytokines IL-6 and TNF-*α* (3.9-4.7-fold) in addition to an increase in the level of oxidative stress [31]. The results of the present study revealed that expression of RIPK1 and phosphorylation of MLKL were significantly upregulated in the myocardium of WT myocardial hypertrophy mice, and the levels of serum IL-6 and TNF-*α* were increased. In RIPK3^−/−^AMP-AngII mice, the expression of RIPK1 was reduced, the levels of phosphorylation of MLKL fell, and the levels of serum IL-6 and TNF-*α* were decreased. The effect of intraperitoneal injection of the RIPK3 inhibitor GSK'872 in the AMP-AngII mice was consistent with RIPK3^−/−^ mice, and the specific pathways of RIPK3-induced myocardial necrosis were associated RIPK1 and MLKL. It is therefore hypothesized that RIPK3 gene knockout and RIPK3 inhibition have the same mechanism in reducing necroptosis in cardiac hypertrophic cells.

CaMKII is a multifunctional serine/threonine-protein kinase [11, 32]; it can be regulated by phosphorylation. As the key protein in excitation-contraction coupling (ECC) and excitation-transcription coupling (ETC) [9, 33], CaMKII is a pleiotropic signaling protein that regulates cardiomyocyte Ca^2+^ circulation, contraction, inflammation, metabolism, gene expression, and cell survival [5]. Recent studies have reported that CaMKII is a substrate of RIPK3 [9, 11]. Mechanistically, RIPK3 constitutes an important upstream kinase of CaMKII, and there are at least two pathways involved in RIPK3-mediated activation of CaMKII. These include direct phosphorylation and indirect ROS-mediated oxidation, which subsequently trigger the formation of mitochondrial permeability transition pores and myocardial necrosis [8, 34], Studies have reported that sustained activation of CaMKII is considered to be a central intracellular trigger for various cardiac diseases [34, 35], there is evidence that sustained activation of CaMKII contributes to a large number of major cardiac diseases, such as heart failure, arrhythmias, and cardiomyopathic sudden death [36–38]. The study presented herein identified that the phosphorylation and oxidation of CaMKII were significantly upregulated in WT mice with cardiac hypertrophy, but were significantly reduced in RIPK3^−/−^ mice. The effect of intraperitoneal injection of the RIPK3 inhibitor GSK'872 in mice in the AMP-AngII group was consistent with that observed in RIPK3^−/−^ mice. It is therefore likely that the CaMKII signaling pathway may be downstream of or a substrate for RIPK3 in cardiac hypertrophy.

CaMKII has four isomers (*α*, *β*, *γ*, and *δ*) that are constitutively expressed in different tissue types with at different rates. The *α* and *β* isoforms are mainly distributed in neurons, whilst *δ* and some *γ* are mainly distributed in cardiomyocytes. CaMKII *δ* is expressed as one of three variants: CaMKII *δ*A, CaMKII *δ*B, and CaMKII *δ*C, after alternative splicing of its exons 14, 15, or 16. Different subtypes of CaMKII *δ* have distinct functions. CaMKII *δ*A mediates the coupling of myocardial excitation and contraction. CaMKII *δ*B is thought to induce pathological cardiac remodeling through phosphorylation of histone deacetylase. CaMKII *δ*C participates in cardiomyocyte apoptosis [38, 39]. The variable shear of CaMKII *δ* is strictly regulated. Once disorder occurs, the expressions of the three variants are unbalanced, leading to dysfunction of myocardial cells and ultimately resulting in heart disease [39, 40]. The experimental results of the present study showed that compared with those of the control group, the levels of CaMKII *δ*A mRNA and CaMKII *δ*B mRNA in the myocardial tissue of the WT (AMP-AngII) mice were reduced, whilst the level of CaMKII *δ*C mRNA was increased; however, in the RIPK3^−/−^(AMP-AngII) group and in the RIPK3 inhibitor GSK'872 group, the levels of CaMKII *δ*A mRNA and CaMKII *δ*B mRNA were upregulated, whilst the levels of CaMKII *δ*C mRNA were decreased. It is suggested that both RIPK3 gene knockout and RIPK3 inhibition can correct the CaMKII *δ* alternative splicing disorder in myocardial tissue of myocardial hypertrophy mice, and their regulatory effects are consistent.

An elevated level of oxidative stress is a central pathophysiological mechanisms contributing to many cardiovascular diseases [41, 42]. Moreover, there is evidence that the production of ROS during myocardial hypertrophy leads to necrosis. In addition, necrosis is considered to exacerbate the production of ROS; in this way, necrosis and ROS form a positive feedback loop [43–45]. Mitochondria are the predominant cellular source of ROS and energy production. If mitochondria become damaged, their oxidative phosphorylation capacity is reduced, which can subsequently result in excessive ROS production and abnormal calcium homeostasis, thereby promoting cardiomyocyte necroptosis [46–48].

Experiments in the study presented herein identified that WT mice with AMP-AngII-induced myocardial hypertrophy had increased serum MDA levels, decreased SOD activity, and T-AOC levels. Furthermore, their mitochondrial ultrastructure was compromised such that mitochondria were swollen and disordered and mitochondrial cristae were shortened. The number of mitochondria in WT hypertrophic mice was reduced, whilst oxidative stress levels were significantly enhanced. In the RIPK3^−/−^ and RIPK3 inhibitor groups, MDA levels in myocardial tissue were reduced, and SOD activity and T-AOC levels were higher, whilst oxidative stress levels were reduced and mitochondrial ultrastructural disturbances were significantly ameliorated. It is suggested that both RIPK3 gene knockout and RIPK3 inhibition can consistently improve the level of oxidative stress in myocardial tissue of myocardial hypertrophy mice and reverse the disorder of mitochondrial ultrastructure.

CaMKII as a new RIP3 molecular substrate. Targeting RIPK3-CaMKII pathway can protect the heart from cardiomyocyte apoptosis or necrosis [8]. Although the animal pathological model used in this study has certain limitations, these findings will help to understand the pathological mechanism of myocardial hypertrophy and provide a scientific basis for the treatment of myocardial hypertrophy, delaying the development of the disease and looking for new therapeutic targets. The commercialized RIPK3 inhibitor GSK'872 is expected to be developed into a new targeted drug for clinical HCM treatment. Therefore, the results of this study have potential transformation and application prospects and have extremely far-reaching significance for clinical diagnosis and/or treatment of cardiovascular diseases.

In conclusion, our current findings reveal that necroptosis has a central role in myocardial necrosis in mice with myocardial hypertrophy. RIPK3 deficiency could ameliorate myocardial injury in mice with cardiac hypertrophy, improve cardiac function, inhibit CaMKII activation, correct the CaMKII *δ* alternative splicing disorder, and reduce necrosis. Collectively, CaMKII activation and necrosis were increased in myocardial hypertrophy in an RIPK3-dependent manner. These findings provided valuable insights into the pathology of myocardial hypertrophy. Our study demonstrated that RIPK3 mediates alternative splicing of CaMKII *δ* in cardiac hypertrophic mice and CaMKII *δ* activation causes disorder of alternative splicing variants, which in turn leads to cardiomyocyte necroptosis. Finally, RIPK3 deficiency and RIPK3 inhibition were highly consistent, both resulted in reduced CaMKII activity, corrected the CaMKII *δ* alternative splicing disorder, alleviated oxidative stress, reduced necroptosis, and reversed myocardial injury caused by angiotensin-induced myocardial hypertrophy in mice. Overall, CaMKII activity and enhanced necroptosis in cardiac hypertrophy are dependent on RIPK3; the RIPK3 inhibitor GSK'872 had a significant protective effect on AMP-AngII-induced cardiac hypertrophy in WT mice. GSK'872 is therefore a promising candidate for the treatment of hypertrophic cardiomyopathy. New targeted drugs for RIPK3 inhibition for clinical targeted therapy of hypertrophic cardiomyopathy could significantly improve clinical outlook for the disease.

## Data Availability

The data used to support the findings of this study are available from the corresponding authors upon request.
